# A Novel Non-Peptidic Agonist of the Ghrelin Receptor with Orexigenic Activity *In vivo*

**DOI:** 10.1038/srep36456

**Published:** 2016-11-07

**Authors:** Elena Pastor-Cavada, Leticia M. Pardo, Dalia Kandil, Cristina Torres-Fuentes, Sarah L. Clarke, Hamdy Shaban, Gerard P. McGlacken, Harriet Schellekens

**Affiliations:** 1 Alimentary Pharmabiotic Centre (APC) Microbiome Institute, University College Cork, Cork, Ireland; 2 Department of Chemistry and the Analytical and Biological Chemistry Research Facility (ABCRF), University College Cork, Cork, Ireland; 3 Department of Anatomy and Neuroscience, University College Cork, Cork, Ireland

## Abstract

Loss of appetite in the medically ill and ageing populations is a major health problem and a significant symptom in cachexia syndromes, which is the loss of muscle and fat mass. Ghrelin is a gut-derived hormone which can stimulate appetite. Herein we describe a novel, simple, non-peptidic, 2-pyridone which acts as a selective agonist for the ghrelin receptor (GHS-R1a). The small 2-pyridone demonstrated clear agonistic activity in both transfected human cells and mouse hypothalamic cells with endogenous GHS-R1a receptor expression. *In vivo* tests with the hit compound showed significant increased food intake following peripheral administration, which highlights the potent orexigenic effect of this novel GHS-R1a receptor ligand.

Cachexia is a metabolic disorder or wasting syndrome, characterised by loss of weight, muscle atrophy, fatigue, weakness and significant loss of appetite, that affects millions of people worldwide, including as many as 80% of people with advanced cancer^1^. In fact, cachexia is seen in the late stages of almost every major chronic illness, affecting 16–42% of people with heart failure, 30% of those with chronic obstructive pulmonary disease and up to 60% of people with kidney disease^1^,^2^. It typically involves extreme weight- and muscle-loss, makes routine activities difficult and increases the risk of infection. For many years it has been overlooked, as physicians and researchers have focused their attention on the primary illness. In 2006 a formal definition emerged, which included a loss of 5% or more of body weight over 12 months, and reduced muscle strength^3^. Now, scientists are increasingly viewing cachexia as a distinct, treatable condition which will undoubtedly lead to novel therapeutic options and improve health and mortality rates.

The regulation of food intake is coordinated in the hypothalamus in the brain. In particular, the arcuate nucleus of the hypothalamus (ARC) is critical for appetite regulation. Many factors are implicated in the hypothalamic regulation of food intake. Among the peripheral peptides, ghrelin and leptin control the central orexigenic and anorexigenic effects respectively^4^,^5^. Other studies on crosstalk between the brain and muscle have assigned an important role to ghrelin in the mechanism of cachexia^6^.

Current treatment options to combat cancer cachexia are limited and mostly palliative. Thus novel strategies and new pharmacotherapies are urgently needed^7^. There is a growing interest in the study of ligands mimicking the appetite-enhancing effects of ghrelin to treat cachexia^8^. Ghrelin acts as an endogenous ligand for the growth hormone secretagogue receptor, or ghrelin receptor (GHS-R1a) and has a plethora of central and peripheral actions including the release of growth hormone from the pituitary gland^9^,^10^,^11^,^12^,^13^,^14^. Ghrelin also plays a key role in the regulation of energy balance by increasing appetite and decreasing energy consumption. This dual effect leads to a positive energy balance, increased food intake and weight gain. Because ghrelin needs parenteral administration and is a peptide with a 30 min half-life, its efficacy in patients is restricted^15^.

Apart from the natural agonist ghrelin, peptide and non-peptide GHS-R1a receptor agonists have been reported^16^,^17^,^18^. In fact, non-peptide mimetics of ghrelin are ideally poised for the development of successful, orally available administration, given the often inhibitory degradation of peptidic based structures^19^, yet reports of non-peptidic GHS-R1a receptor agonists are rare. However, anamorelin (which has a molecular weight of 546.7 g/mol and contains two chiral centres) is currently under development by Helsinn Therapeutics for cancer cachexia and anorexia^20^.

2-Pyrones and their isosteres 2-pyridones are often termed ‘privileged structural motifs’, due to their wide-ranging pharmaceutic properties. In fact many of the key focus-areas of biology have been targeted by 2-pyrone and 2-pyridone containing motifs^21^,^22^,^23^. However as far as we are aware, no report exists of the effect of 2-pyrones or 2-pyridones on GHS-R1a receptor signaling. Thus as part of a wider screening program we sought to test these compounds for GHS-R1a receptor activity.

## Results and Discussion

**Figure 1:**
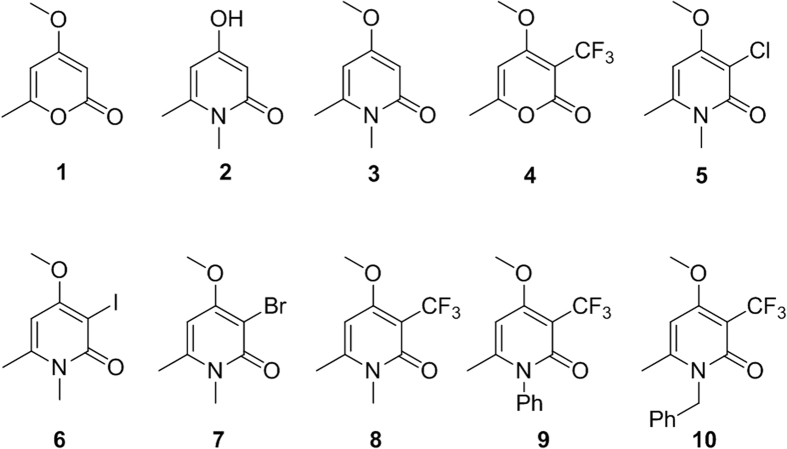
Synthesised 2-pyrones and pyridones.

We initially designed a suite of 2-pyrones and 2-pyridones ranging from simple, commercially available frameworks to novel more decorated structures (Fig. 1).

**Figure 2:**
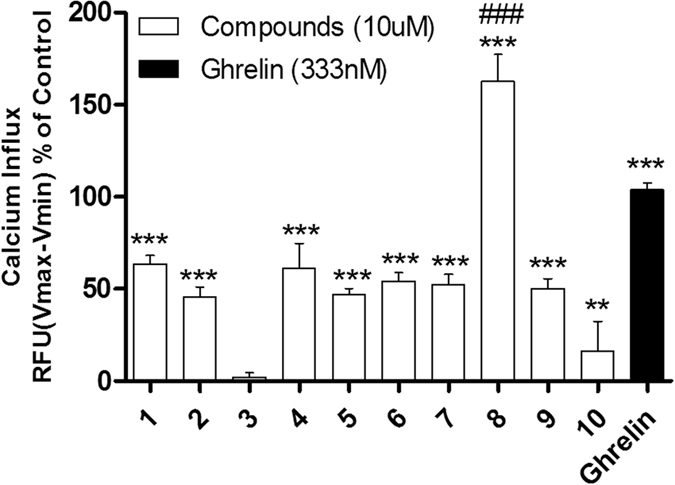
The synthetic small molecules differently affect calcium influx in Hek-GHS-R1a-EGFP cells compared to ghrelin (for details, see SI).

An initial screen of GHS-R1a receptor activation using intracellular calcium mobilisation, which is a measure of downstream GHS-R1a receptor signaling and activation^12^,^24^, was carried out in Hek-GHS-R1a-EGFP cells (human embryonic kidney cells) transfected to express the ghrelin receptor with an enhanced green fluorescent protein tag (EGFP) (Fig. 2). In Phase 1, simple 2-pyrones and 2-pyridones **1**–**3** were tested. Two out of the three showed modest GHS-R1a receptor activity. Modification of the C-3 position (**4**–**8**) dramatically improved the response in the case of pyridone **8**. The introduction of a –CF_3_ group can significantly alter lipophilicity, membrane permeability and oxidative metabolism^25^,^26^,^27^,^28^,^29^. Indeed incorporation of the –CF_3_ group gave dramatically improved activity. Finally, further modification at the N-substituent did not improve calcium influx (**9–10**). Thus, we chose compound **8** for more specific *in vitro* and *in vivo* tests.

**Figure 3:**
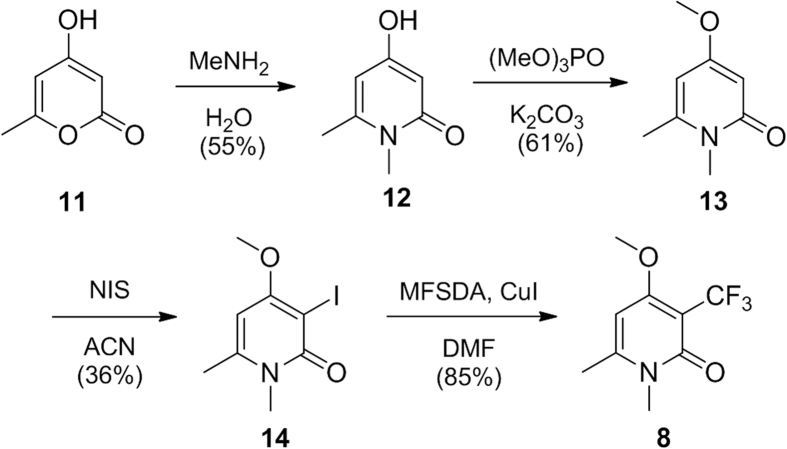
Synthesis of pyridone 8.

Firstly, scale-up (4–5 g) of novel pyridone **8** was achieved via a simple 4 step route, employing a very cheap starting material 4-hydroxy-6-methyl-2-pyrone, **11** (Fig. 3). Refluxing this compound in *N*-methylamine gave the 4-hydroxy substituted pyridone **12** in 55% yield, which can be transformed into the methoxy derivative **13** in 61% yield, and subsequently iodinated selectively at the C-3 position to give **14** in 36% yield.

Methyl 2,2-difluoro-2-(fluorosulfonyl) acetate (MFSDA) was used as the reagent for the trifluoromethylation of pyridone **14** to give novel pyridone **8**^30^,^31^. Before returning to in depth biological studies, the prepared pyridone **8** was deemed not toxic in the two cell lines tested: Hek293a-GHS-R1a and mHypoE-N38 using the resazurin toxicity assay (for details, see SI, Figure S1).

**Figure 4:**
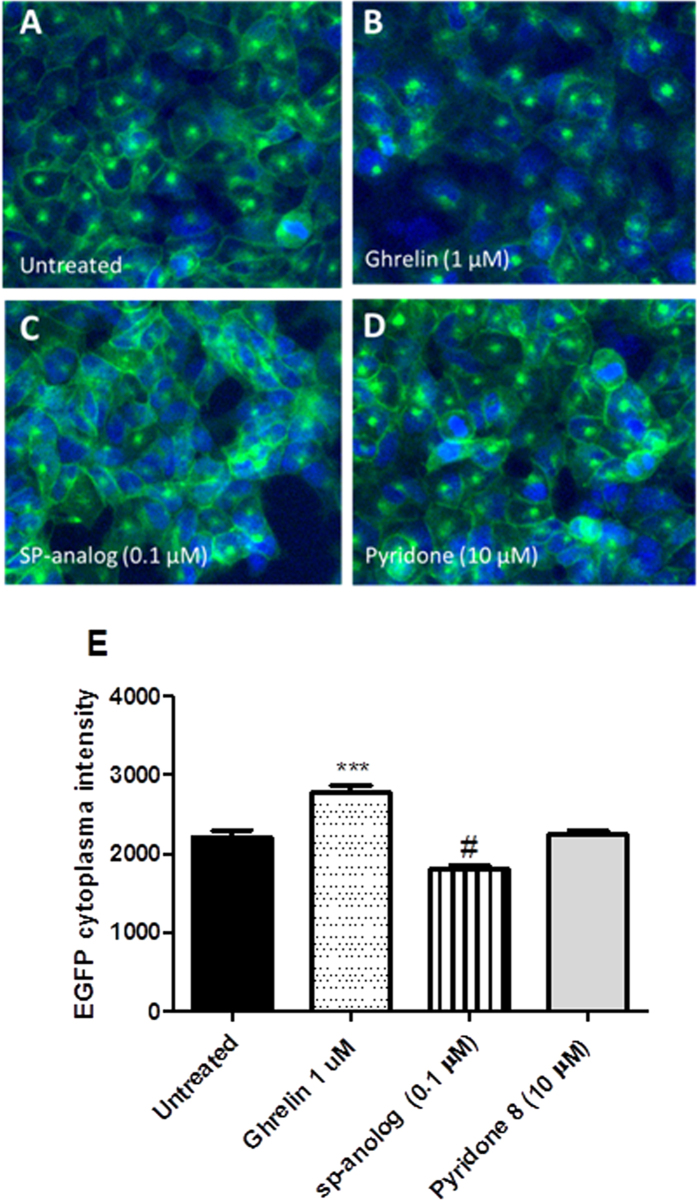
Pyridone **8** does not affect internalisation of the GSH-R1a receptor. Representative images are depicted following different treatments: untreated control (assay buffer), ghrelin (1 μM), [D-Arg1, D-Phe5, D-Trp7,9, Leu11]-substance P (SP-analog) (0.1 μM) and pyridone **8** (10 μM). Graph represents the mean ± SEM of quantified fluorescence intensity (15 pictures per treatment) of perinuclear GHS-R1a-EGFP receptor from a representative experiment (for details, see SI).

In our next set of studies, pyridone **8** was directly compared to the endogenous GHS-R1a receptor agonist, ghrelin and the non-peptide agonist, MK-0677, in the calcium mobilisation assay using Hek-GHS-R1a-EGFP cells (for details, see SI, Figure S2). The hit compound stimulated an intracellular calcium influx, *in vitro*, on cells expressing the GHS-R1a receptor, in a dose dependent manner, with an efficacy of 100% and EC_50_ of 3.1 μM. The pyridone **8** EC_50_ was 35-fold lower compared to endogenous GHS-R1a receptor ligands, ghrelin (EC_50_ 88 nM) and 443-fold lower compared to the EC50 for MK-0677 (EC_50_ 7 nM), a non-peptide GHS-R1a receptor agonist (for details, see SI, Figure S2). The EC_50_s obtained here for ghrelin and MK-0677 are equal to those previously reported by our laboratory as well as others^32^,^33^,^34^,^35^. In contrast, no calcium influx was observed in wild-type Hek cells, showing selectivity for the GHS-R1a receptor. This was further supported by the lack of activity in cells expressing an unrelated serotonin 2C (5-HT_2C_) receptor (for details, see Supporting Information, SI, Figure S3). In addition, pyridone **8**-mediated intracellular calcium mobilisation was enhanced in response to GHS-R1a receptor sensitisation, following pre-treatment with the inverse agonist peptide, [D-Arg1, D-Phe5, D-Trp7,9, Leu11]-substance P (SP-analog) (see SI, Fig. S4A). Inverse agonists, like SP-analog have been shown to decrease the high GHS-R1a constitutive activity leading to an increase of GHS-R1a receptor availability on the membrane. The SP-analog mediated attenuation of the ligand-independent constitutive activity of the GHS-R1a receptor, subsequently leads to enhanced agonist-mediated calcium signaling^36^. Pyridone **8** exposure, following SP-analog pre-treatment, was shown to significantly enhance the GHS-R1a receptor mediated calcium influx at 10 μM (p < 0.001) showing a greater calcium increase than ghrelin control exposure (for details, see SI, Fig. S4A). In addition, pre-treatment with the specific GHS-R1a receptor antagonist, JMV2959, followed by pyridone **8** exposure significantly reduced GHS-R1a receptor mediated calcium influx at 10 μm (p ≤ 0.001) (see SI, Fig. 4B).

The effect of pyridone **8** on GHS-R1a receptor internalisation into endosomes (a characteristic of receptor desensitisation^37^) was then analysed. Both desensitisation and internalisation processes provide essential physiological “feedback” mechanisms that protect against both acute and chronic overstimulation of receptors^38^. Internalisation and trafficking of the GHS-R1a-EGFP receptor was monitored following analysis of EGFP-mediated fluorescence intensity within the cell cytosol (Fig. 4). While the full agonist ghrelin led to a significantly increased GHS-R1a-EGFP receptor internalisation, pyridone **8** exposure did not significantly change receptor trafficking. This suggests a functional selectivity of the pyridone **8** extract towards activation of the calcium signaling, without subsequent GHS-R1a receptor internalisation and desensitisation. In contrast, treatment with the inverse agonist SP-analog did show a significantly decreased EGFP fluorescence intensity within the cytosol, due to blockade of ligand-independent constitutive receptor signaling and consequent increased membrane receptor expression. In any case, this result is informative and indeed suggests that pyridone **8** might ultimately allow for prolonged ghrelineric signaling.

**Figure 5:**
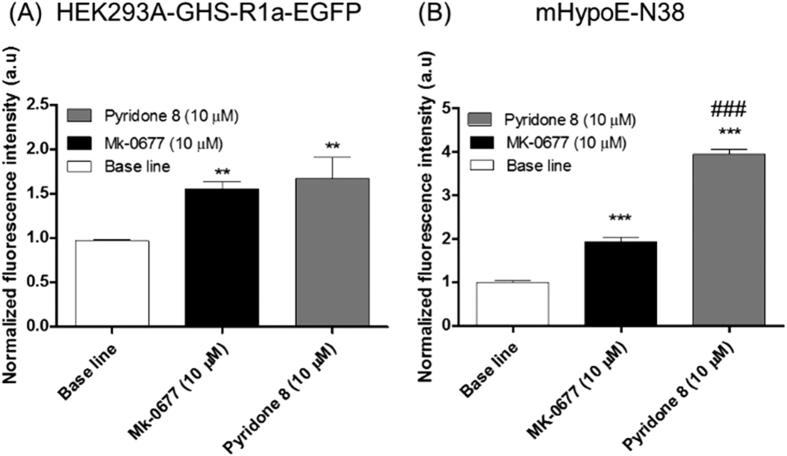
Pyridone 8 activates endogenous GHS-R1a receptor in hypopthalamic neurons. Pyridone **8** and MK-0677 interacting with GHS-R1a receptors in transfected human embryonic kidney (Hek293a) cells (**A**) and embryonic mouse hypothalamic cell line-N38 (**B**) by cell calcium imaging using confocal microscopy (for visualisation, see supplementary videos in SI).

**Figure 6:**
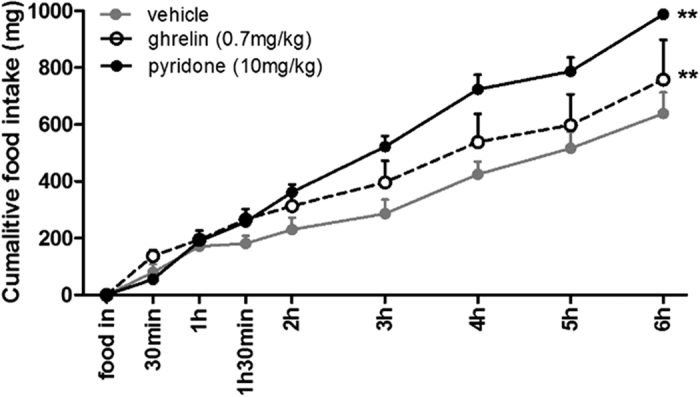
Pyridone 8 increases cumulative food intake *in vivo* compared to vehicle. The GHS-R1a receptor ligand, ghrelin, is used as an orexigenic reference compound (for further details, see SI).

Next, the effect of hit compound **8** on GHS-R1a-receptor mediated intracellular calcium mobilisation using confocal imaging was investigated. We focused especially on a hypothalamic neuronal cell line model of an embryonic mouse, the hypothalamus cell line-N38 (mHypoE-N38). It possesses endogenous levels of GHS-R1a receptor expression (see SI, Figure S5), and would therefore represent a physiologically relevant model to analyse the effects of the 2-pyridone, even predicting its ability to modify hypothalamic neurons *in vivo*. When pyridone **8** was added, the normalised fluorescence intensity was significantly increased compared to baseline and the positive control, MK-0677, a known agonist of the GHS-R1a receptor^39^ (Fig. 5 and for a live imaging videoclip see the SI), indicating the potential of pyridone **8** as a novel agonist for the GHS-R1a receptor. Thus, we conclude that pyridone **8** interacts with the GHS-R1a receptor, as elicited by increased intracellular calcium influx, measured using calcium mobilisation assay and confocal calcium imaging, and therefore acts as full GHS-R1a receptor agonist *in vitro*, without immediate GHS-R1a receptor internalisation and desensitisation. Finally, the biological activity of pyridone **8** was assessed *in vivo* via cumulative food intake measurements in male C57BL/6J mice. A significant acute increase in food intake was observed following intraperitoneal administration of pyridone **8** in *ad libitum* fed mice (Fig. 6 and SI, S6). The endogenous ligand of the GHS-R1a receptor, ghrelin, was used as a reference compound and demonstrated increased food intake, as expected, mainly in the immediate phase after administration. It will be interesting to investigate if the prolonged food intake of pyridone **8** compared to ghrelin is related to an increased GHS-R1a receptor availability, since internalisation and subsequent GHS-R1a receptor desensitisation do not occur following pyridone **8** exposure. In addition, pharmacokinetics and bioavailability of the novel pyridone **8** remain to be investigated. Together, the data confirm that the pyridone **8** acts as a potent GHS-R1a receptor agonist *in vitro* and *in vivo*. The *in vivo* activity of pyridone **8** is certainly worthy of further investigation. This is especially significant given that the GHS-R1a receptor agonists GHRP-6, L-692,429, L-692,585, and MK-0677 have been investigated for growth hormone release and c-fos expression in the hypothalamus^40^, but effects on food intake have not been reported. In fact, we are aware of no other studies reporting non-peptide GHS-Ra receptor agonists with acute orexigenic effects (i.e. within first 2 hours of administration), apart for the novel pyridone **8,** which we demonstrate here to modify food intake *in vivo* at two hours after administration and up to 6 hours. Moreover, the observed long-lasting effect of the orexigenic effect in mice (i.e. up to 6 hours), following intraperitoneal injection of the 2-pyridone, is promising for the development of treatments for eating and metabolic disorders in humans. This is in sharp contrast to the native peptide ghrelin, which has an initial orexigenic effect, following acute peritoneal administration, which tends to tapers off (relative to vehicle) after 2 hours^41^ (and SI, Figure S6), most likely due to its short half-life, and thus restricted efficacy in patients.

In summary, we describe a novel, small, non-peptidic 2-pyridone which acts as a potent GHS-R1a receptor agonist *in vitro*, which translates to an orexigenic effect *in vivo*. In the absence of any FDA approved treatment, the lead compound shows significant potential for use in the treatment of cachexia. Future development will progress in two ways: 1) Structural optimisation (installation of groups at the C3 position of pyrones and pyridones is difficult^42^,^43^,^44^,^45^ and will require the development of new chemistry) and, 2) further selectivity studies (beyond 5HT-2c) to confirm GHS-R1a receptor specificity. However, a very promising molecular template has emerged and the fact that such a simple molecule demonstrates potent ghrelin receptor agonist activity (*in vitro* & *in vivo*), with promising selectivity is remarkable.

## Material and Methods

### Synthesis and Characterisation of compounds 1–10

#### General information

Melting point determinations were performed by the open capillary method and are reported uncorrected. IR spectra were recorded on Perkin-Elmer FT-IR Paragon 1000 spectrophotometer. Liquid samples were examined as thin films interspersed on NaCl plates. Solid samples were dispersed in KBr and recorded as pressed discs. The intensity of peaks were expressed as strong (s), medium (m) and weak (w) and broad (b). ^1^H, ^13^C and ^19^F NMR spectra were recorded at 25 °C in CDCl_3_ at 300, 75 and 282 MHz spectrometer unless otherwise specified, with TMS as the internal standard. Chemical shifts (^1^H, ^13^C and ^19^F) were expressed as parts per million (ppm) positive shift being downfield from TMS; coupling constants (*J*) are expressed in Hertz (Hz). High-resolution mass spectra (HRMS) were obtained on a TOF MS instrument with ESI source. Literature citations are provided for known compounds and representative characterisation data. Analytical thin layer chromatography (TLC) was performed on silica gel 60 F_254_ aluminum plates (Merck). TLC plates were visualized by exposure to short wave ultraviolet light (254 nm, 366 nm). Column chromatography was carried out using 60 Å (35–70 mm) silica. The Microanalysis Laboratory, National University of Ireland, Cork, performed elemental analysis using a Perkin-Elmer 240 and Exeter Analytical CE440 elemental analysers.

**4-Methoxy-6-methyl-2*****H*****-pyran-2-one, 1**


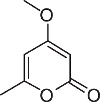
 Compound **1** was prepared according to a known procedure and spectral characteristics were consistent with previously reported data^46^.

**4-Hydroxy-1,6-dimethylpyridin-2(1*****H*****)-one, 2**


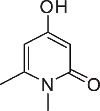
 Compound **2** was prepared according to a known procedure and spectral characteristics were consistent with previously reported data^47^.

**4-Methoxy-1,6-dimethylpyridin-2(1*****H*****)-one, 3**


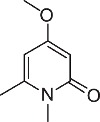
 Compound **3** was prepared according to a known procedure and spectral characteristics were consistent with previously reported data^48^.

**4-Methoxy-6-methyl-3-(trifluoromethyl)-2*****H*****-pyran-2-one, 4**


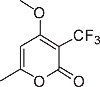
 Compound **4** was prepared according to a known procedure and spectral characteristics were consistent with previously reported data^31^.

**3-Chloro-4-methoxy-1,6-dimethylpyridin-2(1*****H*****)-one, 5**


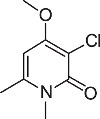
 To a stirred solution of 4-methoxy-1,6-dimethylpyridin-2(1*H*)-one, **3**, (0.500 g, 3.3 mmol) in acetonitrile (10 mL) was added *N*-chlorosuccinimide (0.880 g, 6.6 mmol). The reaction vessel was covered in aluminium foil and resulting mixture allowed to stir at reflux overnight. The solvent was concentrated in vacuo, the resulting solid dissolved in CH_2_Cl_2_ (20 mL) and washed with 5% aqueous sodium thiosulfate (2 × 25 mL), water (25 mL) and brine (25 mL). The organic layer was dried over MgSO_4_ and concentrated in vacuo to yield crude product, which was purified by silica column chromatography to yield compound **5** as a pale yellow solid (0.316 g, 51% yield). m.p. 168–170 °C. IR (KBr) ν_max_: 1649 (amide C=O stretch, s), 1589 (aromatic C=C stretch, s), 1350 (ester C-O stretch, s) cm^−1^; ^1^H NMR (300 MHz, CDCl_3_): δ 2.38 (3H, s, C***H***_***3***_), 3.56 (3H, s, NC***H***_***3***_), 3.92 (3H, s, OC***H***_***3***_), 5.96 (1H, s, C***H***) ppm; ^13^C NMR (75.5 MHz, CDCl_3_): δ 21.4 (***C***H_3_), 32.0 (N***C***H_3_), 56.4 (O***C***H_3_), 105.4 (***C-***Cl), 94.8 (***C***H), 145.1 (CH_3_***C***CH), 160.8 (***C***=O), 161.7 (***C***OCH_3_), ppm; HRMS (ESI) *m/z* calcd for C_8_H_11_NO_2_Cl [(M + H)^+^]: 188.0478, found 188.0470.

**3-Iodo-4-methoxy-1,6-dimethylpyridin-2(1*****H*****)-one, 6**


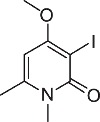
 To a stirred solution of 4-methoxy-1,6-dimethylpyridin-2(1*H*)-one, **3**, (0.362 g, 2.36 mmol) in acetonitrile (15 mL) was added *N-*iodosuccinimide (1.603 g, 4.73 mmol). The reaction vessel was covered in aluminium foil and allowed stir at reflux overnight. On completion, the solvent was concentrated in vacuo, the resulting orange solid dissolved in CH_2_Cl_2_ (20 mL) and washed with 5% aq. sodium thiosulfate (3 × 25 mL). The organic layer was dried over MgSO_4_ and concentrated in vacuo to yield crude product which was purified by silica column chromatography eluting with 70:30 hexane:ethyl acetate to yield compound **6** as a yellow solid (0.238 g, 36%). m.p. 191–193 °C. IR (KBr) ν_max_: 1628 (amide C=O stretch, s), 1585 (aromatic C=C stretch, s), 1342 (ester C-O stretch, s) cm^−1^; ^1^H NMR (300 MHz, CDCl_3_): δ 2.35 (3H, s, C***H***_***3***_), 3.57 (3H, s, NC***H***_***3***_), 3.88 (3H, s, OC***H***_***3***_), 5.86 (1H, s, C***H***) ppm; ^13^C NMR (75.5 MHz, CDCl_3_): δ 21.4 (***C***H_3_), 32.5 (N***C***H_3_), 56.6 (O***C***H_3_), 71.8 (***C-***I), 94.7 (***C***H), 147.6 (CH_3_***C***CH), 162.0 (***C***=O), 166.5 (***C***OCH_3_), ppm; HRMS (ESI) *m/z* calcd for C_8_H_11_NO_2_I [(M + H)^+^]: 279.9835, found 279.9827.

**3-Bromo-4-methoxy-1,6-dimethylpyridin-2(1*****H*****)-one, 7**


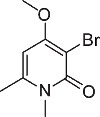
To a stirred solution of 4-methoxy-1,6-dimethylpyridin-2(1*H*)-one, **3**, (0.500 g, 3.3 mmol) in acetonitrile (10 mL) was added *N*-bromosuccinimide (1.180 g, 6.6 mmol). The reaction vessel was covered in aluminium foil and resulting mixture allowed to stir at reflux overnight. The solvent was concentrated in vacuo, the resulting solid dissolved in CH_2_Cl_2_ (20 mL) and washed with 5% aqueous sodium thiosulfate (2 × 25 mL), water (25 mL) and brine (25 mL). The organic layer was dried over MgSO_4_ and concentrated in vacuo to yield crude product, which was purified by silica column chromatography to yield compound **7** as a pale yellow solid (0.345 g, 45% yield). m.p. 170–172 °C. IR (KBr) ν_max_: 1651 (amide C=O stretch, s), 1593 (aromatic C=C stretch, s), 1346 (ester C-O stretch, s) cm^−1^; ^1^H NMR (300 MHz, CDCl_3_): δ 2.35 (3H, s, C***H***_***3***_), 3.54 (3H, s, NC***H***_***3***_), 3.89 (3H, s, OC***H***_***3***_), 5.91 (1H, s, C***H***) ppm; ^13^C NMR (75.5 MHz, CDCl_3_): δ 21.4 (***C***H_3_), 32.2 (N***C***H_3_), 56.4 (O***C***H_3_), 94.8 (***C***H), 95.7 (***C-***Br),146.2 (CH_3_***C***CH), 163.2 (***C***=O), 177.2 (***C***OCH_3_), ppm; HRMS (ESI) *m/z* calcd for C_8_H_11_NO_2_Br [(M + H)^+^]: 231.9973, found 231.9976.

**4-Methoxy-1,6-dimethyl-3-(trifluoromethyl)pyridin-2(1*****H*****)-one, 8**


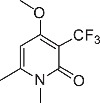
 To a stirred solution of 3-iodo4-methoxy-1,6-dimethylpyridin-2(1*H*)-one, **6**, (0.235 g, 0.84 mmol) and copper(I) iodide (0.192 g, 1.01 mmol) in DMF (4 mL) was added methyl 2,2- difluoro-2-(fluorosulfonyl)acetate (0.13 mL, 1.01 mmol). The reaction mixture was allowed stir at 70 °C for 7 h. On completion, the reaction was cooled to room temperature, diluted with diethyl ether (20 mL) and filtered. The solution was poured into water (20 mL), extracted with diethyl ether (4 × 20 mL) and the combined organic extracts washed with water (3 × 5 mL). The organic layer was dried over MgSO_4_ and concentrated in vacuo to yield crude product which was purified using silica column chromatography eluting with 70:30 hexane:ethyl acetate to yield **8** as a white solid (0.114 g, 61%). m.p. 136–138 °C. IR (KBr) ν_max_: 1651 (amide C=O stretch, s), 1598, 1563 (aromatic C=C stretch, s), 1130 (ester C-O stretch, s) cm^−1^; ^1^H NMR (300 MHz, CDCl_3_): δ 2.40 (3H, s, C***H***_***3***_), 3.47 (3H, s, NC***H***_***3***_), 3.89 (3H, s, OC***H***_***3***_), 5.92 (1H, s, C***H***) ppm; ^13^C NMR (75 MHz, CDCl_3_): δ 21.8 (***C***H_3_), 30.9 (N***C***H_3_), 56.3 (O***C***H_3_), 94.1 (***C***H), 99.9 (q, ^2^*J*C-F = 29.2 Hz, ***C***CF_3_), 124.0 (q, ^1^*J*C-F = 272.8 Hz, ***C***F_3_), 151.4 (CH_3_***C***CH), 160.7 (***C*** = O), 166.4 (***C***OCH_3_) ppm^19^; F NMR (282 MHz, CDCl_3_): δ −57.4 (C***F***_**3**_) ppm; HRMS (ESI) *m/z* calcd for C_9_H_11_F_3_NO_2_ [(M + H)^+^]: 222.0742, found 222.0736; Anal. calcd for C_9_H_10_F_3_NO_2_: C, 48.87; H, 4.56; N, 6.33%. Found: C, 49.09; H, 4.43; N, 6.19%.

**4-Methoxy-6-methyl-1-phenyl-3-(trifluoromethyl)pyridin-2(1*****H*****)-one, 9**


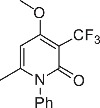
To a round bottomed flask containing 4-hydroxy-6-methyl-1-phenylpyridin-2(1*H*)-one^49^ (0.776 g, 3.86 mmol) and K_2_CO_3_ (0.640 g, 4.63 mmol) was added trimethylphosphate (0.93 mL, 8.01 mmol) and the resulting reaction mixture stirred at reflux overnight. On completion, the reaction was washed with water (15 mL) and extracted with ethyl acetate (3 × 25 mL). The combined organic extracts were dried over MgSO_4_ and concentrated in vacuo to yield crude product as an orange oil which was purified by silica column chromatography eluting with ethyl acetate to yield **4-methoxy-6-methyl-1-phenylpyridin-2(1*****H*****)-one** a pale yellow solid (0.578 g, 70%). m.p. 162–163 °C. IR (KBr) ν_max_: 2968 (alkyl C-H stretch, m), 1650 (amide C=O stretch, s), 1597, 1562 (aromatic C=C stretch, s), 1243 (C-O stretch, m) cm^−1^; ^1^H NMR (300 MHz, CDCl_3_): δ 1.85 (3H, s, C***H***_***3***_), 3.73 (3H, s, OC***H***_***3***_), 5.81 (1H, s, CH_3_CC***H***), 5.85 (1H, d, *J* = 2.5 Hz, C***H***C=O), 7.14 (2H, d, *J* = 7.4 Hz, 2 × C***H***arom.), 7.36–7.48 (3H, m, 3 × C***H***arom.) ppm; ^13^C NMR (75.5 MHz, CDCl_3_): δ 21.5 (***C***H_3_), 55.4 (O***C***H_3_), 95.0 (***C***HC=O), 100.8 (CH_3_C***C***H), 128.3, 128.7, 129.7 (5 × ***C***H arom.), 138.7 (N***C***), 146.0 (CH_3_***C***CH), 165.5 (***C***=O), 167.9 (***C***OCH_3_) ppm; HRMS (ESI) *m/z* calcd for C_13_H_14_NO_2_ [(M + H)^+^]: 216.1025, found 216.1018; Anal. calcd for C_12_H_11_NO_2_: C, 71.63; H, 5.51; N, 6.96%. Found: C, 71.64; H, 5.71; N, 6.77%. To a stirred solution of 4-methoxy-6-methyl-1-phenylpyridin-2(1*H*)-one (0.578 g, 2.69 mmol) in acetonitrile (25 mL) was added *N*-iodosuccinimide (1.208 g, 5.37 mmol). The reaction vessel was covered in aluminium foil and allowed stir at reflux for 12 h. On completion, the solvent was concentrated in vacuo, the resulting brown solid dissolved in CH_2_Cl_2_ (20 mL) and washed with 5% aq. sodium thiosulfate (3 × 20 mL). The organic layer was dried over MgSO_4_ and concentrated in vacuo to give crude product which was purified using silica column chromatography eluting with 50:50 hexane:ethyl acetate to yield **3-iodo-4-methoxy-6-methyl-1-phenylpyridin-2(1*****H*****)-one** as a pale brown solid (0.768 g, 84%). m.p. 222–224 °C. IR (KBr) ν_max_: 1639 (amide C=O stretch, s), 1590, 1518 (aromatic C=C stretch, s), 1226 (ether C-O stretch, s) cm^−1^; ^1^H NMR (300 MHz, CDCl_3_): δ 2.01 (3H, s, C***H***_***3***_), 3.97 (3H, s, OC***H***_***3***_), 5.99 (1H, s, C***H***), 7.16 (2H, d, *J* = 7.6 Hz, 2 × C***H***arom.), 7.41–7.52 (3H, m, 3 × C***H***arom.) ppm; ^13^C NMR (75.5 MHz, CDCl_3_): δ 22.0 (***C***H_3_), 56.8 (O***C***H_3_), 72.2 (***C-***I), 94.7 (***C***H), 127.9, 128.9, 129.7 (5 × ***C***H arom.), 138.9 (N***C***), 147.6 (CH_3_***C***CH), 162.2 (***C***=O), 167.2 (***C***OCH_3_) ppm; HRMS (ESI) *m/z* calcd for C_13_H_13_O_2_NI [(M + H)^+^]: 341.9991, found 341.9984; Anal. calcd for C_13_H_12_O_2_NI: C, 45.77; H, 3.55; N, 4.11%. Found: C, 45.81; H, 3.57; N, 3.77%. To a stirred solution of 3-iodo-4-methoxy-6-methyl-1-phenylpyridin-2(1*H*)-one (0.356 g, 1.04 mmol) and copper(I) iodide (0.239 g, 1.25 mmol) in DMF (4 mL) was added methyl 2,2-difluoro-2-(fluorosulfonyl)acetate (0.26 mL, 1.04 mmol). The reaction mixture was allowed stir at 70 °C for 24 h. On completion, the reaction was cooled to room temperature, diluted with diethyl ether (20 mL) and filtered. The solution was poured into water (20 mL), extracted with diethyl ether (4 × 20 mL) and the combined organic extracts washed with water (3 × 5 mL). The organic layer was dried over MgSO_4_ and concentrated in vacuo to yield crude product as a white solid which was purified by silica column chromatography eluting with 80:20 hexane:ethyl acetate to yield 4-methoxy-6-methyl-1-phenyl-3-(trifluoromethyl)pyridin-2(1*H*)-one, 9, as a pale yellow solid (0.086 g, 29%). m.p. 205–207 °C. IR (KBr) ν_max_: 1658 (amide C=O stretch, s), 1551 (aromatic C=C stretch, s), 1392 (ether CO stretch, s) cm^−1^; ^1^H NMR (300 MHz, CDCl_3_): δ 2.03 (3H, s, C***H***_***3***_), 3.96 (3H, s, OC***H***_***3***_), 6.01 (1H, s, C***H***), 7.16–7.18 (2H, m, 2 × C***H***arom.), 7.44–7.53 (3H, m, 3 × C***H***arom.) ppm; ^13^C NMR (75 MHz, CDCl_3_): δ 22.4 (***C***H_3_), 56.5 (O***C***H_3_), 94.1 (***C***H), 100.5 (q, ^2^*J*C-F = 28.3 Hz, ***C***CF_3_), 123.8 (q, ^1^*J*C-F = 273.0 Hz, ***C***F_3_), 128.0, 129.1, 129.8 (5 × ***C***H arom.), 137.8 (N***C***), 151.5 (CH_3_***C***CH), 160.9 (***C***=O), 167.3 (***C***OCH_3_) ppm; ^19^F NMR (282 MHz, CDCl_3_): δ −57.2 (C***F***_**3**_) ppm; HRMS (ESI) *m/z* calcd for C_14_H_13_NO_2_F_3_ [(M + H)^+^]: 284.0898, found 284.0893.

**1-Benzyl-4-methoxy-6-methyl-3-(trifluoromethyl)pyridin-2(1*****H*****)-one, 10**


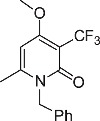
Compound **10** was prepared according to a known procedure and spectral characteristics were consistent with previously reported data^31^.

### Biological Tests: Materials and Methods

#### Compounds

The pyridone **8** was dissolved in assay buffer (1x Hanks balanced salt solution, HBSS, containing 20 mM HEPES) at 10 μM containing 0.3% DMSO and diluted further to desired concentrations. The GHS-R1a receptor agonists ghrelin (SP-GHRL, Innovagen) and MK-0677 (#5272, Tocris), JMV2959 (JMV) (#345888, Calbiochem), as well as the inverse agonist [D-Arg1, D- 102 Phe5, D Trp7,9, Leu11]Substance P (SP-analog) (#1946, Tocris) were prepared as stock solution at several concentrations in assay buffer.

#### Cell culture

Human embryonic kidney cells (Hek293a) (Invitrogen) were maintained in culture in high glucose Dulbecco’s modified Eagle’s medium (DMEM, Invitrogen) containing 10% heat inactivated fetal bovine serum (FBS) (Sigma-Aldrich) and 1% non-essential amino acids (NEAA) (Gibco) at culture conditions (37 °C and 5% CO_2_ in a humidified atmosphere). Hek293a cells were stably transfected with a plasmid construct expressing the human GHS-R1a receptor (Genecopeia, X0963; Accession code, U60179.1) as previously described^35^ and cultured in complete DMEM media, containing 300 ng/μl G418 (Calbiochem) as maintenance antibiotic.

Embryonic mouse hypothalamus cell line N38 (mHypoE-N38) (Cederlane Laboratories) were maintained in culture in high glucose Dulbecco’s modified Eagle’s medium (DMEM, Invitrogen) containing 10% heat inactivated fetal bovine serum (FBS) (Sigma-Aldrich) at culture conditions (37 °C and 5% CO_2_ in a humidified atmosphere).

Cells were grown to a confluence of >85% and subsequently split to a lower density for continued culturing.

#### Resazurin assay

Cytotoxicity of pyridone was determined using the resazurin assay (R&D systems, Inc.) according to manufacturer’s instructions. Resazurin is a blue non-toxic, water soluble, redox-sensitive dye that undergoes a colour change following reduction by viable cells. Absorbance of the colour change is measured at 570 nm. Hek293a-GHS-R1a and mHypoE-N38 cells were seeded in a 96-well microtiter plate at 2.8 * 10^5^ cells/ml (2.8 * 10^4^ cells/well) and maintained for 48 h at culture conditions. For the last 24 h of this time period, media was replaced with serum free DMEM media containing 1% NEAA to mimic calcium mobilization assay conditions. Pyridone **8** was dissolved in saline at 10 μM containing 0.3% DMSO (Sigma-Aldrich). Then, it was centrifuged for 5 min at 2000 rpm and the supernatant was used to analyse its cytotoxicity. Cells were exposed for 4 h to this pyridone comprising 10% resazurin dye. Cell viability was calculated as percentage of control (cells in 1x Hanks balanced salt solution, HBSS) (Gibco), supplemented with 20 mM HEPES (Sigma-Aldrich). Values above 90% were deemed viable and hence compounds used for cell exposure not cytotoxic.

#### Calcium mobilisation assay

G-protein coupled receptor-mediated changes in intracellular calcium (Ca^2+^) were determined using a Flex station II multiplate fluorometer (Molecular Devices). Calcium mobilisation assays were performed according to the manufacturer’s instructions and as previously described^34^,^35^. Briefly, stably transfected Hek293a cells were seeded in black wall, clear bottom 96-well microtiter plates at a density of 2.8 * 10^5^ cells/ml (2.8 * 10^4^ cells/well) and maintained at culture conditions overnight. At cell density of 80–90%, growth media was replaced by serum free DMEM media containing 1% NEAA for a further incubation at culture conditions overnight. After removal of the serum free DMEM media, cells were incubated for 90 min with 25 μl of assay buffer (1x Hanks balanced salt solution, HBSS, containing 20 mM HEPES) and 25 μl of 1x Ca4 dye (Molecular Devices). While it has been determined that 2.5% DMSO is not toxic to cells^34^, the DMSO concentration exposed directly on the cells was no higher than 0.33% in this assay. Fluorescent readings were taken for a total of 80 seconds at 37 °C in flex mode with excitation wavelength of 485 nm and emission wavelength of 525 nm. Addition of ghrelin or a serial dilution of the test compound 8 (25 μl/well) was performed by the Flexstation II after 16 sec during continuous fluorescent measurements for a total of 80 secs. The relative increase in intracellular calcium [Ca^2+^] was calculated as the difference between maximum and baseline fluorescence (Vmax-Vmin) and depicted as percentage relative fluorescent units (RFU) normalized to maximum response (100% signal) obtained with 3.3% FBS, which gives a maximal calcium influx. Background fluorescence was obtained by cells in assay buffer alone and subtracted from RFUs. Exposure to pyridone **8** and endogenous ligand, ghrelin, following pretreatment to the inverse agonist [D-Arg1, D-Phe5, D-Trp7,9, Leu11]-substance P were also carried out. To this end, pretreatment was performed during the 90 min of calcium dye incubation. Responses are considered positive when calcium influx exceeds 20% compared to control. Data were analysed using GraphPad Prism software (PRISM 5.0; GraphPAD Software Inc., San Diego, CA, USA). Sigmoidal dose-response curves were constructed using nonlinear regression analysis with variable slope, excluding values resulting from obvious incorrect pipetting by the Flexstation II.

#### Receptor internalisation assay

The potential of compounds to internalise the GHS-R1a receptor expressed with a C-terminal enhanced green fluorescent protein (EGFP) tag was analysed using an internalisation assay. Hek-GHS-R1a-EGFP cells were seeded in a poly-L-lysine (Sigma-Aldrich) coated 96-well microtiter plate at 3 * 10^4^ cells per well and incubated for 48 h at culture conditions. For the last 16–24 h of this time period media was replaced with serum free DMEM media. Cells were treated with pyridone **8** solution at 10 μM containing 0.3% DMSO for 1 h at 37 °C. The cells were fixed with 4% paraformaldehyde in phosphate buffer saline (PBS) for 20 min, washed three times with PBS. Ligand induced GHS-R1a-EGFP trafficking was imaged on the GE Healthcare IN Cell Analyser 1000 (GE Healthcare, Buckinghamshire, UK) in PBS. Ghrelin was used as positive control. In addition, treatment with the inverse agonist, [D-Arg1, D-Phe5, D-Trp7,9, Leu11]-substance P (SP-analog), which inhibits GHS-R1a receptor constitutive activity and enhances GHS-R1a receptor membrane expression, was also carried out. Receptor trafficking was quantified using the InCell Analyzer Developer Toolbox V1.6 software (GE Healthcare, Buckinghamshire, UK) and expressed as EGFP fluorescence intensity (I) in the cell cytoplasm. Briefly, a total of 15 pictures were analysed using the automated software across 3 independent images. Considering that the GHS-R1a-EGFP receptor accumulates in the perinuclear space after internalisation, a target segmentation based on the fluorescence intensity of perinuclear space was used. The average fluorescence intensity was expressed as the mean ± SEM. Data were analysed and depicted using GraphPad Prism software (PRISM 5.0; GraphPAD Software Inc.).

#### Confocal microscopy technique

HEK-GHS-R-1a-EGFP cells and mHypoE-N38 were seeded in poly-L-lysine coated 35 mm dishes plate (MatTek) at a density of 15 * 10^4^ cells/ml and maintained for ~24 h at culture conditions (37 °C and 5% CO_2_ in a humidified atmosphere). After 24 hours in culture cells were incubated for 40 minutes at 37 °C in a 4 uM solution of Fluo 4 AM (Molecular probes) for HEK-GHS-R-1a-EGFP cells and in a 8 uM solution of Fluo 4 AM for mHypoE-N38 cells. After removal of the dye, cells were washed twice with PBS and 2 ml of assay buffer (1x Hanks balanced salt solution, HBSS, containing 20 mM HEPES) were added to the cells which were stored in the dark at 37 °C until visualisation under microscope.

Each dish was mounted on the stage, under culture conditions (37 °C and 5% CO_2_ in a humidified atmosphere) for stable recording for longer period of time using laser scanning confocal fluorescent microscopy (FV 1000 Confocal System; Olympus). Fluorescence was visualised on an inverted microscope (CKX41; Olympus) setup with a sensitive XM10 camera (C-BUN-F-XM10-BUNDLE) with an infrared cut filter, mercury burner (USH-103OL), and fluorescence condenser (CKX-RFA; Olympus). Green Flourescence of tagged GFP protein was monitored with excitation wavelength of 485 nm and emission wavelength of 525 nm. The same configuration was used to monitor calcium mobilisation signals. For image acquisition 500 usecond/pixel was used. Cells within a single field of view were imaged over 1 minute period in absence of compound (line base) with a 10-s shuttered interval between each image. When each compound was added the field of view was imaged over a 2 minutes period, with a 1-s shuttered interval between each image. Fluorescent intensity changes (from t 0) were measured and expressed as area under the curve and normalised against baseline.

#### Cumulative food intake

A total of 24 male C57BL/6J mice (Harlan laboratories), 8 weeks of age on date of arrival, were group housed in standard holding cages at the animal care facility of University College Cork. The holding room temperature (21 ± 1 °C) and humidity (55 ± 10%) were controlled under a 12 h light/dark cycle (lights on 7.00 AM, lights off 7.00 PM. Water and food (2018S Teklad Global 18% Protein Rodent Diet, Harlan laboratories) were available ad libitum for the duration of the study unless otherwise stated. Mice underwent habituation to experimental conditions over three consecutive days prior to experimentation in which they were single housed for up to 4 hours following intraperitoneal injection of saline (0.9% NaCl). Following this cumulative food intake studies, with eight animals per group, were performed based on protocols described in previous studies^41^,^50^. Briefly, on the day of experimentation animals were single housed for 30 minutes without access to food or water. Following this, mice subsequently received intraperitoneal injections of vehicle (saline, 1% DMSO), ghrelin (200 nmol/kg) or pyridone **8** (10 mg/kg in 1% DMSO). The dose of ghrelin was based on previous studies^50^,^51^. Dose of the unknown, pyridone **8**, was based on similar experiments with small molecules^52^,^53^,^54^,^55^. After 20 minutes a pre- weighed chow food pellet was placed into the cage and was subsequently weighed at regular time intervals (30 min, 1 h, 1.5 h, 2 h, 3 h, 4 h, 5 h and 6 h). At the end of the experiment the mice were placed back into their original group cages. Data were analysed using GraphPad Prism software (PRISM 5.0; GraphPAD Software Inc.). All experiments were approved by the Animal Experimentation Ethics Committee at University College Cork and were carried out in accordance with the relevant guidelines - 200 European Directive 2010/63/EU.

#### Statistical analysis

Statistical analysis was performed using SPSS software (IBM SPSS statistics 22). Normality of the data as well as homogeneity of variance were tested by Shapiro-Wilk test and Levene test respectively. For *in vitro* data analysis a one-way ANOVA followed by Bonferroni post hoc test (when comparing all groups against each other) or Dunnett test (when comparing against a control group) was performed if data was distributed normal and variance was homogenous for all groups. A non-parametric multiple comparisons Kruskal-Wallis test was performed if data was not normal, followed by, where appropriate, Mann-Whitney U tests for individual comparisons. Cumulative food intake was analysed using a general linear model of repeated measures, followed by a one-way ANOVA and multiple comparison tests (LSD post hoc) to analyse specific time points. Level of significant in all analysis was α = 0.05 and all tests were two-tailed test. All graphs represent the mean ± SEM from N independent experiments (see figure legends for N details for each experiment). Statistical significances are subsequently depicted as follows: *indicating p ≤ 0.05, **indicating *p* ≤ 0.01 or ***indicating *p* ≤ 0.001.

### Biological Tests: Results

#### Cytotoxicity assay for pyridone 8

The potential cytotoxicity of pyridone **8** solution was analysed to test its suitability for cell culture studies. This was assessed by the resazurin assay, which is a widely used method to analyse viability of bacteria and mammalian cells^56^. Viability of Hek cells (Figure S1A) and N-hypothalamus cells are depicted (Figure S1B), calculated as percentage of control (cells in assay buffer, HBSS/20 mM HEPES). Cells were exposed for 4 h to pyridone **8** solution at 10 uM concentration. No cytotoxic effects were observed (<4%), showing a cellular viability around 100% with respect to the control, which makes the pyridone **8** a safe, suitable compound for the cellular calcium mobilisation assay.

#### Screen of compounds 1–10 on calcium influx of GHS-R1a receptor on Hek-GHS-R1a-EGFP cells

Compound-mediated intracellular calcium mobilisation via the GHS-R1a receptor in Hek-GHS-R1a-EGFP cells was analysed (Fig. 2). Pyridones tested consisted of simple 2-pyrones and 2-pyridones (**1–3**), pyridones with modified C-3 position (**4–8**) and modification at the N-substituent (**9–10**). Pyridone **8** showed increased calcium influx versus other synthetic compounds and negative control (serotonin). Pyridone **8** was superior in GHS-R1a receptor mediated calcium influx, also compared to the endogenous ligand ghrelin (p ≤ 0.001), and was selected for further *in vitro* and *in vivo* tests.

#### Specific GHS-R1a receptor *in vitro* activation by pyridone 8

GHS-R1a receptor modulation following pyridone **8** exposure was analyzed in the calcium mobilisation assay and compared with the natural agonist ghrelin across cells not expressing the GHS-R1a receptor (wild-type Hek cells, Hek293A wt) and in cells expressing an unrelated G-protein receptor, the serotonin 2C (5-HT_2C_) receptor. No calcium influx was observed in wild-type Hek cells (Hek293A wt, not expressing the GHS-R1a receptor) when exposed to pyridone **8** and ghrelin (Figure S2). In addition, neither pyridone **8** nor the agonist ghrelin, were able to activate the 5-HT_2C_ receptor, indicating specificity for the GHS-R1a receptor (Figure S2).

Exposure of Hek cells stably expressing the GHS-R1a receptor (Hek-GHS-R1a-EGFP) to a serial dilution of pyridone **8** demonstrated a GHS-R1a receptor-mediated calcium influx in a concentration dependent manner (Figure S3). Pyridone **8** activated the GHS-R1a receptor with an EC50 of ~2 uM and efficacy of 100%, behaving as a fullGHS-R1a receptor agonist.

The pyridone **8** mediated calcium mobilisation following pre-treatment with the GHS-R1a receptor specific inverse agonist peptide, [D-Arg1, D-Phe5, D-Trp7,9, Leu11]-substance P (SP-analog) was analysed (Figure S4a). Pyridone **8** exposure following SP-analog pre-treatment did significantly enhance the GHS-R1a receptor mediated calcium influx at 10 μM (p ≤ 0.001) showing a similar calcium increase compared to ghrelin control (333 nM) exposure following SP-analog preincubation. In addition, pre-treatment with the specific GHS-R1a receptor antagonist, JMV2959, followed by pyridone **8** exposure significantly reduced GHS-R1a receptor mediated calcium influx at 10 μm (p ≤ 0.001) (Figure S4b).

#### GHS-R1a receptor internalisation by pyridone 8

The GHS-R1a receptor has a high constitutive activity in the absence of ligand. Following ligand-mediated receptor activation a desensitisation process occurs in order to protect the cell against receptor overstimulation^57^. This process of desensitisation is a consequence of a combination of the uncoupling of the receptor from heterotrimeric G proteins and its internalisation from membrane to intracellular compartments into endosomes^57^. Then, the receptor is marked for degradation or recycling back to the membrane and is a hallmark of receptor activation^38^. Following exposure to agonists, like ghrelin, the GHS-R1a receptor also internalises after signaling, which desensitises the receptor and attenuates further GHS-R1a receptor signaling. Internalisation of the GHS-R1a receptor was investigated in Hek cells stably expressing the receptor as an EGFP-tagged fusion construct. GHS-R1a receptor trafficking could be monitored following analysis of EGFP fluorescent translocation from the cellular membrane into endosomes within the cytosol (untreated, Fig. 4A). Clear internalisation of the GHS-R1a receptor could be observed after treatment with the endogenous agonist ghrelin at 1 μm (Fig. 4B). After treatment with the pyridone **8**, no changes in GHS-R1a receptor internalisation compared to untreated cells was observed (Fig. 4D). In contrast, treatment with inverse agonist SP-analog resulted in increased GHS-R1a-EGFP receptor translocation to the membrane with respect to untreated cells (Fig. 4C) and consequently a significant decreased EGFP fluorescence intensity in the cytosol (p ≤ 0.001) (Fig. 4E). In conclusion, pyridone **8** did not affect GHS-R1a-EGFP fluorescence translocation, which means that GHS-R1a receptor desensitization does not occur and the GHS-R1a receptor remains available on the membrane for continued signaling (Fig. 4E).

#### Pyridone 8 activity on HEK-GHS-R1a-EGFP cells and embryonic mouse N-hypothalamus cell line-N38 using confocal microscopy

Cell calcium imaging using confocal microscopy was carried out to know more about the pyridone **8** interacting with ghrelin receptors in HEK-GHS-R1a-EGFP cells and in the embryonic mouse hypothalamus cell line-N38 (mHypoE-N38), which is an immortalised neuronal cell line with endogenous levels of GHS-R1a receptor expression. MK-0677 (a known agonist of ghrelin) was used as a positive control^58^,^59^,^60^,^61^.

Figure 5A shows a significant increase in the GHS-R1a receptor activation as measured by normalised fluorescence intensity on Hek-293A-GHS-R1a-EGFP for both compounds (p ≤ 0.01 for MK-0677 and pyridone **8**). Figure 5B shows a significant increase in calcium mobilisation in the mHypoE-N38 cell line for both compounds (p ≤ 0.001 for MK-0677 pyridone **8**). Baseline shows normalised fluorescence in the absence of any compound. Finally, Fig. 5B also shows a significant enhanced response (p ≤ 0.001) of the pyridone **8** with respect to MK-0677 in embryonic mouse hypothalamic immortalised neurons (mHypoE-N38).

#### Cumulative food intake

The effect of pyridone 8 on cumulative food intake was investigated in male C57BL/6J mice (n = 8 vehicle, n = 7 ghrelin and n = 8 pyridone 8) at the start of the light cycle (Fig. 6). Pyridone 8 (10 mg/kg in saline containing 1% DMSO), ghrelin (200 nmol/kg in saline containing 1% DMSO) or vehicle (saline, 1% DMSO) were administered to ad libitum fed mice via intraperitoneal (IP) injection 20 minutes before placement of pre weighed food pellets in the cages. Cumulative food intake was measured at regular time intervals over six hours. Repeated measures analysis revealed a clear significant effect of time F (7, 140) = 128.5; p < 0.000 and a significant interaction of treatment × time F (14, 140) = 3.518; p < 0.000 compared to vehicle (Fig. 6) with post hoc LSD analysis showing an overall significant effect of treatment X time of ghrelin and pyridone 8 (p = 0.023 and p = 0.004 respectively). Post hoc LSD analysis of cumulative food intake showed an overall significant effect of acute exposure to 200 nmol/kg ghrelin treatment at individual time points 30 minutes to 2 hours. Specifically, ghrelin significantly increased food intake compared to vehicle and pyridone 8 at 30 minutes (p = 0.022 and p = 0.004, respectively, Figure S5A) and compared to vehicle at 1 h 30 min (p = 0.04, data not shown) and 2 hours after injection (p = 0.23 and 0.011 respectively, Figure S5B). Ghrelin’s orexigenic effect is known to be immediate and then taper off, as previously reported in our laboratory, as well as in other groups^33^,^41^,^51^. Post hoc LSD analysis of cumulative food intake showed an overall significant effect of exposure to 10 mg/kg pyridone 8 treatment at individual time points from 2 hours onwards. A significant higher cumulative food intake was observed with pyridone 8 compared to the vehicle control after 2 h following food placement until the end of experiment at 6 h, at all-time points (p ≤ 0.01 for 2, 4, and 6 hours, Figure S5B, S5C, S5D).

## Additional Information

**How to cite this article**: Pastor-Cavada, E. *et al.* A Novel Non-Peptidic Agonist of the Ghrelin Receptor with Orexigenic Activity *In vivo. Sci. Rep.* **6**, 36456; doi: 10.1038/srep36456 (2016).

**Publisher’s note**: Springer Nature remains neutral with regard to jurisdictional claims in published maps and institutional affiliations.

## Supplementary Material

Supplementary Information

Supplementary Video 1

Supplementary Video 2

Supplementary Video 3

Supplementary Video 4
