# Supplementary material for: A Novel Non-Peptidic Agonist of the Ghrelin Receptor with Orexigenic Activity In vivo
Source: Sci Rep. 2016 Nov 7;6:36456. doi: 10.1038/srep36456 (PMC5098229; doi:10.1038/srep36456)
Supplement: Supplementary Information [file srep36456-s1.doc]

A Novel Non-Peptidic Agonist of the Ghrelin Receptor with Orexigenic Activity in vivo

Elena Pastor-Cavada^1^, Leticia M. Pardo^2^, Dalia Kandil^3^, Cristina Torres-Fuentes^1^, Sarah L. Clarke^2^, Hamdy Shaban^3^, Gerard P. McGlacken^2,^*, and Harriet Schellekens^1,3^*

^1^Alimentary Pharmabiotic Centre (APC) Microbiome Institute, University College, Cork, Ireland

^2^Department of Chemistry and the Analytical and Biological Chemistry Research Facility (ABCRF), University College Cork, Cork, Ireland

^3^Department of Anatomy and Neuroscience, University College, Cork, Ireland

*E-mail: h.schellekens@ucc.ie

*E-mail:g.mcglacken@ucc.ie

Table of contents

| NMR spectra for pyridone **8** | S3 |
| --- | --- |
| Biological Tests |  |
| Figure S1. Cell viability of pyridone **8** | S6 |
| Figure S2. Pyridone **8** concentration response curve | S7 |
| Figure S3. Pyridone **8** Pyridone **8** and ghrelin effects on calcium influx | S8 |
| Figure S4. Pyridone **8** effects on cells pre-treated with GHS-R1a ligands  Figure S5. Expression levels of GHS-R1a receptor mRNA  Figure S6. In vivo cumulative food intake  Supplementary Video Legends  Further information on Figures in main manuscript | S9  S10  S11  S12  S13 |
| References | S14 |
|  |  |
|  |  |

**NMR spectra for pyridone 8**

**4-Methoxy-1,6-dimethyl-3-(trifluoromethyl)pyridin-2(1*H*)-one, 8**

**Biological tests**


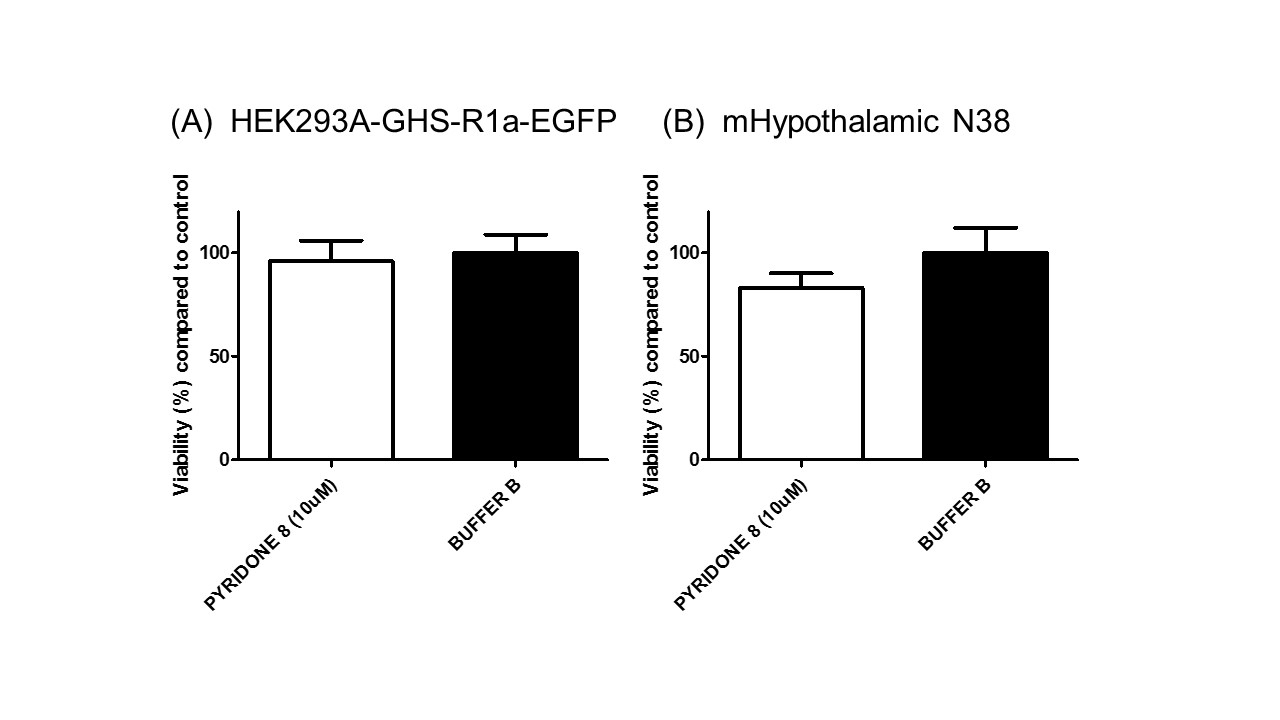


**Figure S1. Cell viability of pyridone 8**. No cytotoxicity is observed. Results are expressed as percentage of viability with respect to the control (assay buffer). Graphs represent the mean ± SEM of triplicate samples from one representative assay.

**Figure S2. Pyridone 8 concentration response curve**. Graph represents the mean ± SEM from triplicate values from two independents experiments for pyridone **8** and the two GHS-R1a ligands, ghrelin and MK0677. Intracellular calcium increase was depicted as a percentage of maximal calcium increase as elicited by control (3.3% FBS). EC_50_s are indicated in the figure legend in order of potency.

**Figure S3. Pyridone 8 and ghrelin effects on calcium influx in Hek293a (wild type), Hek-GHS-R1a-EGFP and Hek-5HT_2C_-EGFP cells**. Graphs represent the mean ± SEM from four independents experiments with each sample performed in triplicate. Intracellular calcium increase was depicted as a percentage of maximal calcium increase as elicited by control (3.3% FBS). No intracellular calcium mobilization in Hek293A wt or 5-HT_2C_-EGFP expressing cells. Significant increases in intracellular calcium mobilization following exposure to pyridone **8** (white bar) or ghrelin (black bar) in the Hek-GHS-R1A-EGFP cell line compared to the wild type line and the Hek-5HT_2C_-EGFP cell line are depicted by +++ p≤0.001. Significant differences of pyridone **8** compared to ghrelin within each cell line are depicted by ** p≤0.01 (Kruskal-Wallis followed by Mann-Whitney U).


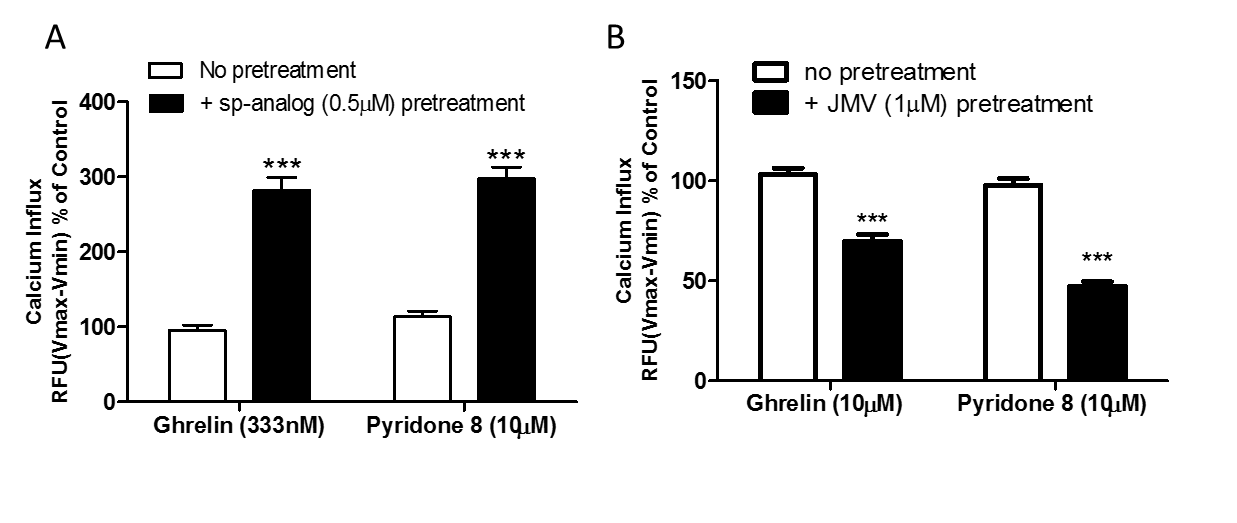

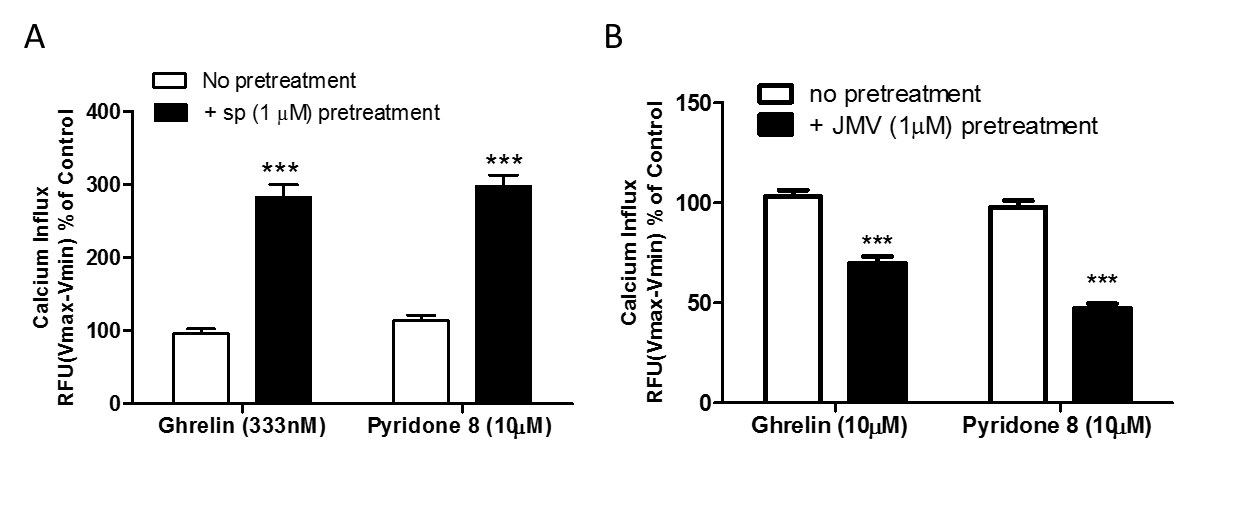


**Figure S4. Pyridone 8 effects on cells pre-treated with GHS-R1a receptor ligands.** Hek-GHS-R1a-EGFP cells were pre-treated with and without the GHS-R1a inverse agonist peptide [D-Arg1, D-Phe5, D-Trp7, 9, Leu11]-substance P (SP-analog) (A) or with the GHS-R1a receptor antagonist JMV2959 (JMV) (B). Graph represents the mean ± SEM from three (A) and two (B) independent experiments with each sample performed in triplicate. Intracellular calcium increase was depicted as a percentage of maximal calcium increase as elicited by control (3.3% FBS). ***p≤0.001 compared with no [D-Arg1, D-Phe5, D-Trp7,9, Leu11]-substance P pre-treatment (One-way ANOVA and Bonferroni’s post hoc).

**Figure S5 Expression levels of GHS-R1a receptor mRNA in mHypothalamic N38 cells***.*  Total RNA was isolated using the Absolutely RNA® Miniprep kit (Stratagene, La Jolla, USA) according to manufacturer's instructions. Quantitative PCR (Q-PCR) was carried out using 6 carboxy fluorescein (FAM™) dye-labeled TaqMan® MGB probes supplied by Applied Biosystems™ to mouse specific GHS-R1a receptor while using β-Actin as an endogenous control. Cycle threshold (Ct) values were normalised using β-Actin and transformed using the 2−ΔCt method.^1^

**
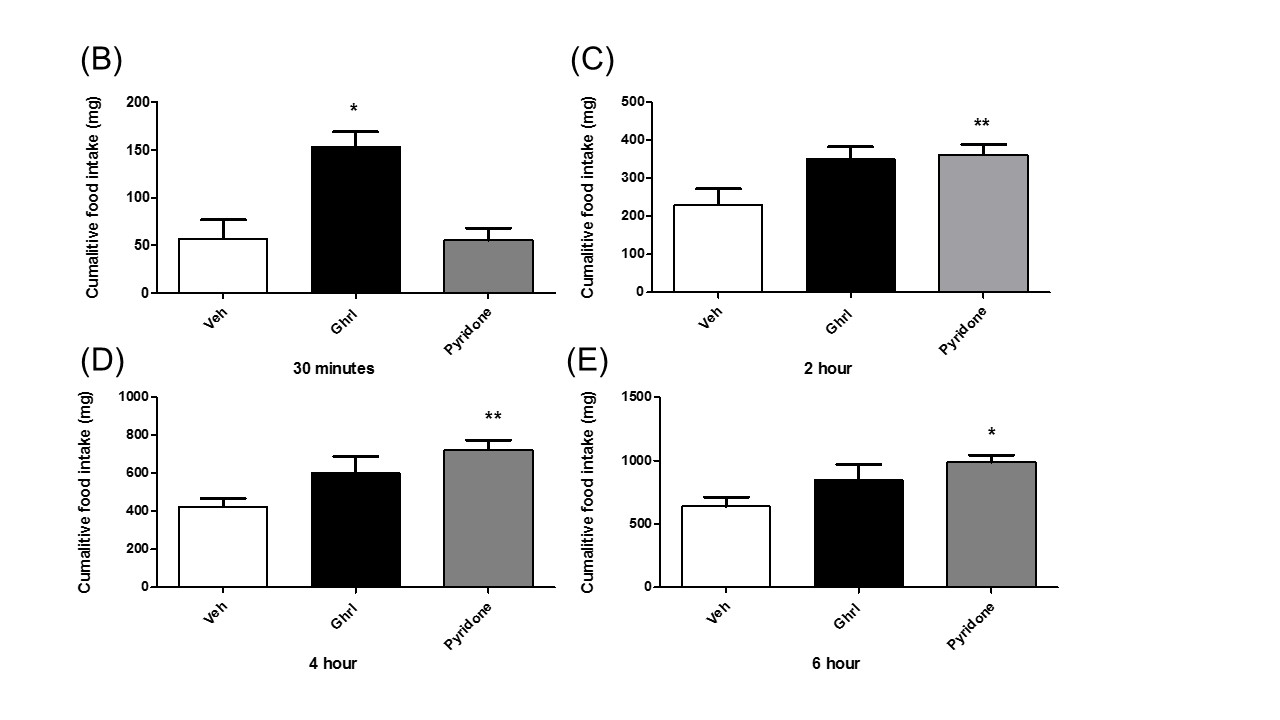
**

**Figure S6. In vivo cumulative food intake.** Cumulative food intake over 6 hours (see **Figure 6**, main article) and at time points 30 min, 2 h, 4 h and 6 h (**Figure S5A, S5B , S5C and S5D**) are depicted for ad libitum fed male C57Bl/6J mice following intraperitoneal administration of pyridone **8** (10 mg/kg) , ghrelin (0.7 mg/kg) or vehicle (saline, 1% DMSO). Repeated measures analysis followed by LSD post hoc test showed a significant effect of pyridone **8** on cumulative food intake when compared to vehicle. Ghrelin, the endogenous ligand for the GHS-R1a receptor is used here as a reference compound significantly increasing acute food intake. Significant increase in food intake is depicted as * p<0.05, ** p<0.01.

**Supplementary Video Legends**

**MK0677_ mHypoE-N38 (Control)**

mHypoE-N38 neurons exposed to the GHS-R1a receptor agonist MK0677. An increase in calcium influx as indicated by an increase in intracellular green fluorescence from the calcium indicator Fluo 4 AM is observed at 5s.

**MK0677_Hek-GHS-R1a-EGFP (Control)**

Hek-GHS-R1a-EGFP cells exposed to the GHS-R1a receptor agonist MK0677. The GHS-R1a-EGFP receptor can be observed on the cell membrane as green fluorescence. An increase in calcium influx as indicated by an increase in intracellular green fluorescence from the calcium indicator Fluo 4 AM is observed at 1s.

**Pyridone 8_ mHypoE-N38**

mHypoE-N38 neurons exposed to pyridone **8**. An increase in calcium influx as indicated by an increase in intracellular green fluorescence from the calcium indicator Fluo 4 AM is observed at 4s.

**Pyridone 8_ Hek-GHS-R1a-EGFP**

Hek-GHS-R1a-EGFP cells exposed to pyridone **8**. The GHS-R1a-EGFP receptor can be observed on the cell membrane as green fluorescence. An increase in calcium influx as indicated by an increase in intracellular green fluorescence from the calcium indicator Fluo 4 AM is observed at 1s.

**Further information for Figures in main manuscript**

**Further information for Figure 2, main article:**

**Figure 2. The synthetic small molecule compounds differently effect calcium influx in Hek-GHS-R1a-EGFP cells compared to ghrelin**. Graphs represent the mean ± SEM from three independents experiments with each synthetic compound (**1-10**) performed in triplicate. Intracellular calcium increase was depicted as a percentage of maximal calcium increase as elicited by control (3.3% FBS). Statistical significant increases in GHS-R1a-mediated calcium mobilisation elicited by pyridone 1-10 and ghrelin are depicted as ** p≤0.01 and *** p≤0.001 compared to blanc. Significance compared to endogenous control, ghrelin, is depicted as ### p≤0.001 (Kruskal-Wallis followed by Mann-Whitney U).

**Further information for Figure 4, main article:**

**Figure 4. Pyridone 8 effects on internalisation of the GSH-R1a receptor**. Hek cells stably expressing the GHS-R1a receptor as a C-terminal EGFP fusion protein were visualised using the IN Cell Analyser 1000 (GE Healthcare) following different treatments: untreated (**A**), ghrelin (**B**), [D-Arg1, D-Phe5, D-Trp7,9, Leu11]-substance P (SP-analog) (**C**) and pyridone **8** (**D**) at the indicated concentrations for 1 h at 37 °C. (**E**) Graph represents the mean ± SEM of quantified fluorescence intensity (15 pictures per treatment) of perinuclear GHS-R1a-EGFP receptor from a representative experiment. Significant increased internalisation is depicted as *** p≤0.001, and significant decreased internalisation is depicted as #p≤0.05 with respect to internalisation obtained from untreated cells (ANOVA followed by Dunnett).

**Further information for Figure 5, main article:**

**Figure 5. Pyridone 8 and MK-0677 activate the GHS-R1a receptor as measured by cell calcium imaging using confocal microscopy.** Graph represents the mean ± SEM from three (A) or two (B) independents experiments on Hek-GHS-R1a-EGFP cells and mouse hypothalamic immortalised cells, respectively. Significant increases of normalised fluorescence intensity by MK-0677 and pyridone are depicted as ** p≤0.01, *** p≤0.001 with respect to base line and ### p≤0.001 compared to MK-0677 (One-way ANOVA and Bonferroni’s post hoc).

**References**

1. Livak, K. J. & Schmittgen, T. D. Analysis of Relative Gene Expression Data Using Real-Time Quantitative PCR and the 2^-ΔΔC^_T_ Method. *Methods* **25,** 402–408 (2001).
